# Ubiquity and Diversity of Human-Associated *Demodex* Mites

**DOI:** 10.1371/journal.pone.0106265

**Published:** 2014-08-27

**Authors:** Megan S. Thoemmes, Daniel J. Fergus, Julie Urban, Michelle Trautwein, Robert R. Dunn

**Affiliations:** 1 Department of Biological Sciences and W. M. Keck Center for Behavioral Biology, North Carolina State University, Raleigh, North Carolina, United States of America; 2 North Carolina Museum of Natural Sciences, Raleigh, North Carolina, United States of America; 3 California Academy of Sciences, San Francisco, California, United States of America; 4 Department of Entomology, North Carolina State University, Raleigh, North Carolina, United States of America; Fordham University, United States of America

## Abstract

*Demodex* mites are a group of hair follicle and sebaceous gland-dwelling species. The species of these mites found on humans are arguably the animals with which we have the most intimate interactions. Yet, their prevalence and diversity have been poorly explored. Here we use a new molecular method to assess the occurrence of *Demodex* mites on humans. In addition, we use the 18S rRNA gene (18S rDNA) to assess the genetic diversity and evolutionary history of *Demodex* lineages. Within our samples, 100% of people over 18 years of age appear to host at least one *Demodex* species, suggesting that *Demodex* mites may be universal associates of adult humans. A phylogenetic analysis of 18S rDNA reveals intraspecific structure within one of the two named human-associated *Demodex* species, *D. brevis.* The *D. brevis* clade is geographically structured, suggesting that new lineages are likely to be discovered as humans from additional geographic regions are sampled.

## Introduction

Many organisms live on us and in us. Fewer than 10% of the cells in our bodies are actually our own. Most of these organisms are bacteria, but we are also colonized by multicellular species including fungi [1], intestinal worms [2] and ectoparasites, such as lice [3]–[5], with nearly 2000 pathogen and parasite species alone known from human bodies [6]. Among the more enigmatic of the multicellular species that live on humans, as well as on other mammals, are mites of the genus *Demodex* (reviewed in [7]), which are common on human faces and other parts of the body [8], [9]. While these mites are well known to dermatologists, ophthalmologists, and veterinarians and have been the subject of study for 172 years (reviewed in [10]), their ubiquity, diversity and evolution are poorly understood. For example, *Demodex* have not been sampled from the vast majority of mammal species, including those that seem very likely to host *Demodex* mites, such as chimpanzees and gorillas. Nor have most human populations been sampled for these mites.

Two species of *Demodex, D. brevis* (Akbulatova 1963) and *D. folliculorum* (Simon 1842), have been described from the human body. In general, *Demodex* live mostly within hair follicles. Biopsies of skin cross-sections reveal *D. folliculorum* to inhabit the area of the follicle above the sebaceous gland, where they appear to ingest cell contents [11]. *D. brevis*, on the other hand, primarily inhabits the sebaceous glands associated with vellus hairs [11], typically at densities of just one to a few mites per gland. With approximately 5 million hair follicles spread across the body [12] and more than 7 billion humans on Earth, the total habitat area available to these mites is immense. Methods used to collect *Demodex* mites from humans include biopsy, the cellophane tape method (placing tape on the face to stick to the mites), scraping areas where mites are likely to reside, and plucking eyelash and eyebrow hairs. Based on the visual observation of mites collected from healthy individuals by these methods, it appears that approximately 3–55% of humans harbor *Demodex*, with most studies falling in the range of 10–20% [8], [13]–[16]. However, because these mites may occur in patches around the body, as in dogs [17], and all existing collection methods sample just small patches of skin (and even incompletely sample those patches), it is difficult to know to what extent the absence of mites in a sample equates to the absence of mites on the body. Intriguingly, in postmortem studies, mites appear to be present on all adult cadavers (reviewed in [10]). The ubiquity of mites on cadavers might indicate they are universally present on living, adult humans but missed by current sampling methods. Alternately, conditions in which cadavers are found might facilitate colonization by mites and, in doing so, artificially inflate estimates of their incidence.

Even less well understood than the proportion of people (or for that matter, other mammals) that host *Demodex* mites is the diversity of those mites. While two species of human-associated mites have been formally named, they were named based on morphological characters alone [18], [19]. Given that *Demodex* mites inhabit restrictive, specialized environments (hair follicles), some aspects of their morphology, including their small size (∼100–200 µM) and general elongate appearance, could reflect convergent evolution among distinct lineages or species groups which would only be discerned by examination of non-morphological data, e.g. by DNA sequence-based differences. A recent study of human *Demodex* species found genetic differences in the mitochondrial CO1 gene between mite populations that inhabit the eyelashes versus mite populations that inhabit the skin [20]. In addition, studies of another human-associated parasite, lice (*Pediculus humanus*), have found strong genetic structure between geographic lineages [4], [5], [21]. Geographic structure among human-associated *Demodex* lineages is expected, given that these mites are more intimately associated with the body than lice and seemingly less mobile, yet the minimal data that exist have not yet recovered such variation [22]. Conversely, if *Demodex* lack strong geographic structure, it suggests the movement of mites among humans must occur very frequently (perhaps even with social greeting rituals) and across large geographic distances.

Only recently have molecular studies begun to consider *Demodex* mites. Existing phylogenies and estimates of molecular divergence include very limited sampling of *Demodex* species, are based on few genetic markers, and include only minimal geographic representation. The DNA sequences that have been obtained from human-associated *Demodex* species come almost exclusively from China (*D. folliculorum* and *D. brevis*) and Spain (*D. folliculorum)* [20], [22]. Studies based on the 16S rRNA gene (16S rDNA) find little variation within *D. folliculorum* and show no geographic structure between samples from China and Spain [22]. However, no molecular data have been considered from *D. brevis* outside of China, and low genetic variation observed for human-associated *Demodex* in previous phylogenies [22] may reflect insufficient sampling rather than the actual genetic diversity of *Demodex* mites.

Here we test a new molecular approach to detect the presence of mites on human bodies and assess the proportion of individuals in one population colonized by mites. We then use phylogenetic reconstruction based on the nuclear 18S rRNA gene (18S rDNA) to better understand the diversity of these mites.

## Materials and Methods

### Ethics Statement

Participants were sampled by project staff at outreach events. Prior to sampling, each participant was verbally informed about the goals of the project and the sampling protocol. All participants were provided and signed a written Informed Consent form. All human *Demodex* sampling procedures and the participant Informed Consent form were approved by North Carolina State University's Institutional Review Board for the Protection of Human Subjects in Research (IRB), Approval No. 2966.

### (a) Sample collection

All sample collections were performed in Raleigh, NC at either the North Carolina Museum of Natural Sciences or North Carolina State University. Each participant was gently scraped with a metal laboratory spatula along the creases of the nose and over the surrounding cheek area. The facial habitats were chosen based on their high levels of sebum production and ease of pore expression. In addition, Bonnar *et al*. (1993) found the greatest abundance of mites in the cheek area among rosacea patients [23]. Mineral oil was typically applied to the sampled area to facilitate mite removal. After collection, the sebum was moved to a drop of mineral oil on a cover slip fragment where it was inspected to note the presence or absence of visually identifiable mites within the sample. Regardless of the presence or absence of observed mites the entire cover slip fragment with the sebum and mineral oil was transferred to a 1.5 ml microcentrifuge tube and maintained in −20°C for subsequent DNA extraction.

### (b) DNA Extraction and PCR

DNA was extracted from the sebum of individual participants, regardless of the presence or absence of an observed mite, using a Qiagen DNeasy Blood & Tissue kit. We followed the manufacturer's supplementary insect protocol, without the initial grinding step. The samples were incubated overnight at 56°C with 180 µl of ATL buffer and 20 µl proteinase K. The final elution step was performed with 150 µl of elution buffer warmed to 56°C.

We used either OneTaq (NEB) or TaKaRa Ex Taq (Clontech), which possess proofreading functions, for all PCR reactions to reduce polymerase induced sequence errors. We designed the primers by aligning all available *Demodex* 16S rDNA or 18S rDNA sequences across the same genes from several other mites and from humans. In an attempt to design primers that were likely to be unbiased with regards to *Demodex* and have a low affinity for the hosts' DNA, we selected priming sites near the 5′ and 3′ ends of most available *Demodex* sequences that were highly conserved among these mites, yet that were unlikely to amplify these genes from humans. The 16S rDNA primer sequences used were 5′-GGTATTTTGACTGTGCTAAGG-3′ and 5′-AAAARCCAACATCGAGGTA-3′, which amplify the region from nucleotide 26 to 358 of *D. folliculorum* sequence FN424245.1. The PCR cycling conditions for 16S rDNA were 94°C for 1 min, followed by 40 cycles of 94°C for 20 s, 47°C for 30 s, 72°C for 1 min and a final 72°C extension for 5 min. The 18S rDNA primers were 5′-GTTGAKCCTGCCAGTAGTCA-3′ and 5′-GTCTGAAGACCTCACTAAATC-3′, which amplify the region from nucleotide 7 to 1688 of *D. folliculorum* sequence JF784006.1. The PCR cycling conditions for 18S rDNA were 94°C for 1 min, followed by 40 cycles of 94°C for 20 s, 45°C for 30 s, 72°C for 2 min and a final 72°C extension for 5 min.

**Figure 1:**
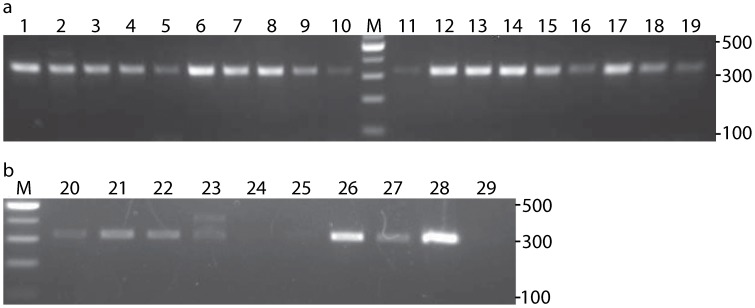
PCR based screen for presence of *Demodex* 16S rDNA in samples with no visually identifiable mites. Lanes labeled 1–29 represent samples from single individual participants. Lanes labeled M represent 100 bp molecular weight size markers. (a) PCR products indicate the presence of *Demodex* DNA in 100% of the screened samples from individuals over the age of 18. (b) PCR products indicate the presence of *Demodex* DNA in 70% of the screened samples from individuals 18 years of age.

The 16S rDNA PCR products were separated on 2% agarose gels to assess presence or absence of mite DNA within a sample. Non-specific amplification of human 16S rDNA occasionally occurred but was easily discernible as an approximately 100 bp larger product (see Figure 1B, lane 4). For this analysis, a set of 19 individuals over 18 years of age and a second set of ten individuals 18 years of age were used. Several 16S rDNA PCR reactions were also sequenced to verify the specificity of the primers. However, data from this gene was not sequenced for most individuals, because this sequence was rather short (∼325 bp) and did not contain many phylogenetically informative sites (i.e., two phylogenetically informative sites exist among our 16S rDNA sequences and the *D. folliculorum* sequences available on GenBank).

The 18S rDNA PCR products were sequenced from four individuals and used for phylogenetic analyses. We chose 18S rDNA for these analyses as this PCR works well with very little incident of non-specific bands (see Figure 1A). Furthermore, the transfer of mtDNA between closely related species has been frequently observed [24]–[26]. By using the nuclear 18S rDNA, we hope to decrease the likelihood of introgression obscuring population or species variation. All sequences were submitted to GenBank (Table 1).

**Table 1:** *Demodex* mite species identification based on 18S rDNA gene sequence.

Host ID	Putative Species	Accession #	Length			Host Sex	Residence
**127**	*D. folliculorum*	KF745876		1636		M	USA
**127**	*D. brevis*	KF745877		1645		M	USA
**127**	*D. brevis*	KF745878		1646		M	USA
**127**	*D. brevis*	KF745879		1007*		M	USA
**127**	*D. brevis*	KF745880		1636		M	USA
**141**	*D. folliculorum*	KF745881		1636		F	USA
**141**	*D. brevis*	KF745882		1646		F	USA
**141**	*D. brevis*	KF745883		1643		F	USA
**141**	*D. brevis*	KF745884		1011*		F	USA
**141**	*D. brevis*	KF745885		1497*		F	USA
**176**	*D. folliculorum*	KF745886	1636		F		Brazil
**176**	*D. folliculorum*	KF745887	1636		F		Brazil
**176**	*D. folliculorum*	KF745888	1636		F		Brazil
**315**	*D. folliculorum*	KF745889		1636		F	Brazil
**315**	*D. folliculorum*	KF745890		1636		F	Brazil
**315**	*D. brevis*	KF745891		1646		F	Brazil
**315**	*D. brevis*	KF745892		1646		F	Brazil

The putative species assignment, GenBank accession number, and sequence length (bp) for each 18S rDNA gene sequence is listed, along with the ID, sex, and country of residence of the hosts. *Indicates partial sequences for which high-quality sequence data was not available for a portion of an amplified fragment.

### (c) Sequencing and Phylogenetic Analysis

Because our faces have the potential to harbor many thousands of individual *Demodex* mites, we expect remnants of these mites to be present in our pores and on the surface of our faces, making the clean isolation of *Demodex* DNA from a single mite difficult. Thus, we presume that each of our scrapings is likely to harbor DNA from multiple mites. To obtain sequences from single copies of 18S rDNA from individual mites, we cloned the 18S rDNA PCR products using TOPO TA Cloning Kits (Invitrogen). We picked and sequenced a minimum of five colonies from each person sampled in this study to get a sense of the diversity within an individual host. The resulting sequences were aligned with *Demodex* sequences available on GenBank using MAFFT v7 [27], with the E-INS-i algorithm, and checked by eye for best alignment. All GenBank sequences are named according to the species names given in GenBank; however, due to the current state of *Demodex* systematics some sequences are likely improperly designated (particularly dog-hosted species), leading to paraphyly of some taxa. The 18S rDNA sequence from a mite species, *Neochelacheles messersmithi*, in the same superfamily as *Demodex*, Cheyletoidea, was included as an outgroup for phylogenetic analysis.

To obtain estimates of genetic divergence between 18S rDNA sequences of all taxa included for phylogenetic analysis, Kimura 2-parameter distances (K2P) [28] and total number of nucleotide differences were calculated using MEGA v5 [29]. Genetic distances were calculated for all pairwise sequence comparisons as well as intra- and interspecific means.

Phylogenetic analyses were conducted using maximum likelihood (ML) and Bayesian inference (BI). Under both methods, gaps in the alignment were treated as missing data. jModelTest 2 [30] was used to determine the best-fitting model for the 18S rDNA data set. Using the corrected Akaike information criterion [31], the TIM2+ I + G model (with two rates of transitions and two rates of transversions) was selected as the best-fitting model for these data [32]. ML analysis was conducted using GARLI 2.0 for Windows [33]. Ten independent search replicates were run under the TIM2+ I + G model, with each replicate run for 100,000 generations. Bootstrap support values for nodes on the ML topology were computed with GARLI by running 1000 bootstrap replicates. The Bayesian analysis was conducted with MrBayes 3.2 [34]. Two independent runs were performed for 50 million generations, each with four chains (three heated and one cold), uninformative priors, and trees sampled at intervals of 1000 generations. Stationarity was determined by examining standard deviation of split frequencies between the two runs for convergence and examination of average potential scale reduction factor (PSRF). Of the 50,000 trees sampled in each run, the first 10,000 trees were discarded as burn-in and the remaining trees were used to construct a 50% majority rule consensus tree. Because the standard deviation of split frequencies was observed to drop and remain below 0.01 by 1,500,000 generations (i.e., 1500 sampled trees), our burn-in value of 10,000 was chosen to ensure that trees were sampled well after runs had reached convergence. The harmonic mean of likelihoods was estimated for post burn-in trees using the *sump* command in MrBayes. We assigned putative species sources for new sequences based solely on phylogenetic distance of previously reported species.

## Results

Based on the observation of visually identifiable mite specimens within our samples, the prevalence of mites in adults was 14% (n = 253), in line with previous studies [8], [13]–[16]. However, we were able to extract *Demodex* 16S rDNA from 100% of adults over the age of 18 (Figure 1A; Mean age: 37±10.4 years, n = 19). Molecular evidence suggests *Demodex* prevalence is much higher than recognized through visual observation alone. Our results are in line with postmortem studies that find *Demodex* mites present on all adult cadavers (reviewed in [10]).

Based on the observation of intact specimens in samples of young adults 18 years of age, mites were found on only 5.88% (n = 51). Of the ten 18 year olds we examined further for *Demodex* 16S rDNA, we amplified 16S rDNA PCR products from only seven samples (Figure 1B). Thus while 100% of adults in our sample hosted *Demodex* mite 16S rDNA, the prevalence and/or detectability in younger individuals appears lower (70%).

**Figure 2:**
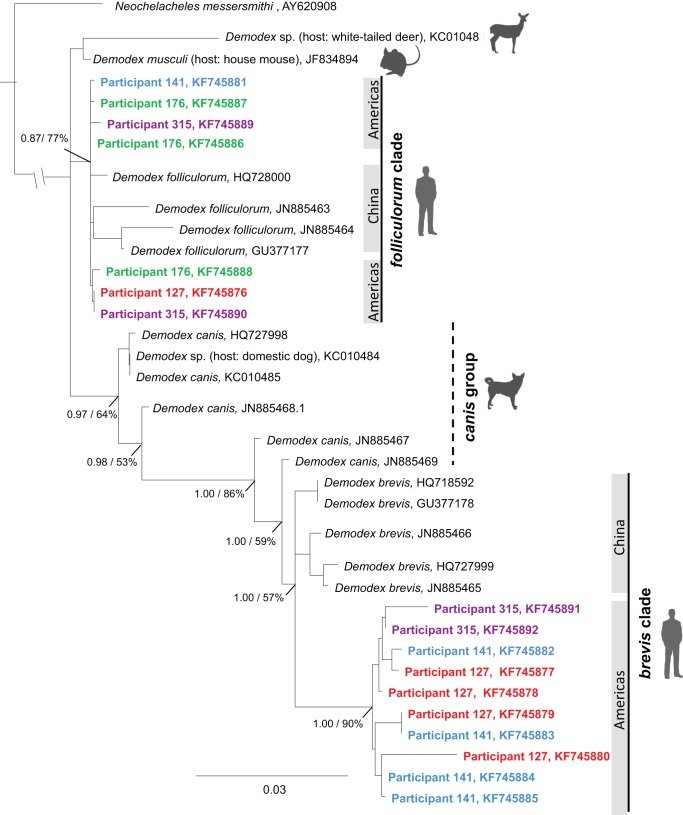
Maximum likelihood (ML) phylogeny of mites based on 18S rDNA sequences. Support values (Bayesian posterior probabilities/ML bootstrap support) given next to major nodes in the topology. Scale bar indicates the number of substitutions per site. Icons indicate mite host.

For phylogenetic analyses, we amplified, cloned, and sequenced *Demodex* 18S rDNA from four individual humans from whom we identified 17 unique *Demodex* 18S rDNA sequences (Table 1). These sequences reflect the presence of multiple mites within a given sample, even if we assume the presence of sequencing error and potential variation among 18S rDNA copies within the genome. We combined these sequences with previously published *Demodex* 18S rDNA sequences, representing at least 5 species from 4 mammalian hosts (human: *D. brevis* and *D. folliculorum*, dog: *D. canis*, mouse: *D. musculi*, and white-tailed deer: *D.* sp.) and an additional mite outgroup, *Neochelacheles messersmithi*, from the same superfamily as *Demodex*, Chelyetoidea (Figure 2). Our alignment comprised 1664 bp for 35 sequences (see Material S1 for alignment). The ML analysis yielded a tree with the best score of –ln  = 4887.29 (see Material S2 for ML tree file). The Bayesian analysis yielded a 50% consensus tree with harmonic mean of likelihood  = −4976.76 (see Material S3 for Bayesian tree file). The average standard deviation of split frequencies of sampled trees  = 0.00119, and the PSRF of sampled trees  = 1.000. Phylogenetic analyses conducted with ML and BI yielded largely congruent topologies; minor incongruencies were restricted to placement of sequences with extremely short internodal branch lengths within the *D. folliculorum* clade and as such do not influence our interpretation. The ML topology is shown in Figure 2, with Bayesian posterior probabilities and ML bootstrap support values depicted adjacent to the major nodes of interest.

As evident in our phylogenetic results, we found substantial genetic diversity among (up to 0.065 K2P distance, up to 20 nucleotide substitutions (nts)) and within *Demodex* species (up to 0.032 K2P, up to 10 nts) (Table S1). Several of our sequences fit within a relatively well-supported *D. folliculorum* clade within which we find low genetic diversity (0.002 K2P, up to 2 nts) even though the individuals sampled included humans from North and South America and sequences from GenBank for individuals from China. Greater diversity is present within the *D. brevis* clade (up to 6.5 K2P, up to 10 nts). Multiple lineages of *D. brevis* appear to be present even on individual humans (within participant diversity: 0.006–0.007 K2P, 2–2.16 nts). However, the greatest diversity was among geographically distinct human populations (up to 0.032 K2P distance between American and Chinese sequences, 10 nts). Existing sequences of *D. brevis* sampled from humans in China resolve as a monophyletic clade sister to a New World clade composed of samples acquired for this study.

## Discussion

Here we tested 29 people for the presence of *Demodex* mites and found that mites were much more common than expected in comparison to methods that rely solely on the visual confirmation of whole mite specimens taken from living humans. When we sampled individuals using traditional approaches, our results were similar to those of the many previous morphologically based studies [8], [13]–[16]; 14% of individuals over the age of 18 had visually observed mites. But when we identified the presence of mites based on the amplification of *Demodex* DNA, we found that every adult over 18 years of age and 70% of 18 year olds had detectable *Demodex* 16S rDNA in the collected sebum of facial samples. Though it is possible *Demodex* 16S rDNA could be found on the face of an individual without mites, the likelihood that we detect such transferred DNA in our limited sampling area would be low. Moreover, if intact *Demodex* 16S rDNA were present in the environment at high enough levels to produce the results we see in adults, we would expect to see the same results among the 18 year olds, which we do not.

Little is known about the transmission of mites among humans. Recent studies find that many symbiotic microbes are passed directly from mother to offspring during breast-feeding [35] or during birth (especially if birth is vaginal) [36], [37], and dogs acquire their *Demodex* mites as nursing pups [38]. In light of this, the same means of mite transmission seems possible in humans, supported by the fact that in one study, *Demodex* mites were found in 77% of nipple tissue from mastectomies [39]. Yet that we found mites on all adults but only 70% of 18 year olds, suggests that perhaps mite colonization does not strictly occur vertically, from parent to child. These results are in line with earlier morphological (largely postmortem) studies in which mites were found to be more prevalent on adults than on children (reviewed in [10]). Mites could be more ubiquitous on children than noted in postmortem studies or herein but at levels or in locations that make the mites difficult to detect even with the use of molecular approaches. One study of *Demodex* mites on Tokelau islanders found that mites were present on a greater number of children than on adults [40]. These conflicting findings highlight our limited understanding of how and when mites move onto and among human bodies.

Overall, we found the genetic variation of 18S rDNA within the genus *Demodex* comparable (up to 0.065 K2P) to the level of variation found among other genera within Acari (0.00–0.056 K2P; Ticks: Ixodidae) [41] (Table S1). This diversity suggests *Demodex* is a relatively old genus and even that the divergence between the two named human-associated species, *D. brevis* and *D. folliculorum*, might be relatively ancient. Within *Demodex*, *D. folliculorum* and *D. brevis* exhibit contrasting levels of intraspecific genetic diversity. *D. folliculorum*, which can be found living superficially within pores, show very little variation in the 18S rDNA sequence data we generated (mean of 0.002 K2P, up to 2 nts).

In comparison to *D. folliculorum*, *D. brevis* exhibited higher genetic diversity, not only between mites from the Americas and those from China (up to 0.032 K2P, up to 10 nts) but also among mites collected from the same individual human (0.005–0.009 K2P, 1.6–4.0 nts). Sequences of 18S rDNA from different *D. brevis* samples taken from the same face (of participant 141, Figure 2) exhibited more genetic variation (0.006 K2P, 4 nts) than those of *D. folliculorum* taken from Chinese and North and South Americans (mean 0.002 K2P). The diversity of *D. brevis* 18S rDNA found on individual humans suggests that not only do all adult humans have *Demodex* mites but that colonization is likely to occur more than once.

The Chinese *D. brevis* samples in GenBank and our newly generated samples from the Americas each form monophyletic clades with a relatively deep divergence between them (mean 0.021 K2P, 6.5 nts). The distance between the two *D. brevis* clades suggests strong geographic isolation among populations of *D. brevis.* Based on sequence divergence, these two populations are as different as are many congeneric species and subspecies. The 18S rDNA variation found between these two geographic populations is similar, for example, to that found between subspecies of parasitic lice, the head louse and body louse (*Pediculus humanus capitis* and *Pediculus humanus humanus*) [5]. *D. brevis* can be found more deeply embedded in sebaceous glands below the skin surface, in comparison to *D. folliculorum* that lives more superficially in the hair follicles. These contrasting habitat preferences may lead to more frequent transmission of *D. folliculorum* than of *D. brevis*, thus resulting in greater reproductive isolation and geographic structure in populations. However, given our limited geographic sampling, we expect the *Demodex* topology to change as samples from other regions are integrated.

The evolutionary history of the two human-associated *Demodex* species is, at best, poorly understood. *D. folliculorum* was described by Simon in 1842, and as late as 1933, all human *Demodex* were regarded as one, albeit variable, species [42], [43]. It was only in 1963 that *D. brevis* was distinguished from *D. folliculorum* and described as a separate, but closely related, species [18]. Yet de Rojas *et al*. (2012) have demonstrated that interpreting variation in the morphology of the two human-associated *Demodex* mite species is problematic, even when interpreted in light of molecular (16S rDNA) sequence data [20]. The closest relatives for both human-associated species, *D. folliculorum* and *D. brevis,* remain unknown and are likely to remain unknown until these mites are much better sampled from other primates and mammalian hosts in general. Of the described *Demodex* species, only 13 have been sampled for molecular data and included in phylogenetic analyses. In addition, given that there are over 5000 species of mammals and as of yet, some mammals (such as humans, dogs, and cats) appear to host more than one *Demodex* species, any existing phylogeny represents a minute fraction of the possible species diversity of the genus. *Demodex* are generally considered to be species specific, which would suggest there might be as many as 10,000 *Demodex* species on living mammals if there are two host specific mites per mammal species. Obviously, this estimate depends both on the ubiquity of *Demodex* mites among mammal species and on their true host specificity, both of which are poorly known.

Our phylogeny indicates that the two human-associated mite lineages do not share a recent common ancestor and likely have separate evolutionary histories of transmission to humans. The 18S rDNA sequence does not resolve the sister group to *D. folliculorum*, but places a paraphyletic group of dog-associated mites as the closest relative to *D. brevis.* The dog mite sequences included here were all acquired from GenBank and are primarily labeled *D. canis*. Yet, there are 3 morphologically distinct *Demodex* species that have been described from dogs (*D. canis, D. injai*, and *D. cornei*) and the molecular delimitation of these dog-associated species is not clear [44]. It seems likely that the sequences labeled *D. canis* included here may actually represent multiple dog-hosted *Demodex* species. Phylogenetic estimates based on 16S rDNA also find that dog-hosted *Demodex* mites share a recent common ancestor with a human-associated species, though in this case *D. folliculorum* and *D. brevis* are both more closely related to goat-associated mites, *D. caprae* [45]. The known habitat of *D. canis* is deep within the pores and is most similar to that of *D. brevis.* It is tempting to posit that *D. brevis* may have colonized humans from wolves during their domestication but any such assertion would be premature. Until other primate species are sampled, the mystery of whether humans acquired *Demodex* mites from our ape/hominid ancestors or through other means such as our interactions with domesticated mammal species will remain.

## Supporting Information

Table S1 **Pairwise distances between 18S rDNA sequences from** ***Demodex*** **species.** Lower left  =  Kimura 2-parameter distances; Upper right  =  number of nucleotide differences.
(XLSX)

Material S1 ***Demodex*** **18S rDNA sequence alignment.**
(FAS)

Material S2 **Tree file for the maximum likelihood (ML) tree.**
(TRE)

Material S3 **Tree file for the Bayesian inference (BI) tree.**
(TRE)
